# Prostate cancer follow-up costs in Germany from 2000 to 2015

**DOI:** 10.1007/s11764-021-01006-w

**Published:** 2021-03-01

**Authors:** Thomas Michaeli, Daniel Michaeli

**Affiliations:** Fifth Department of Medicine, University Medical Centre Mannheim, Medical Faculty Mannheim, Heidelberg University, Mannheim, Germany

**Keywords:** Prostate cancer, Follow-up, Budget impact, Cost, Health insurance

## Abstract

**Purpose:**

The main objective of this study is to estimate and evaluate 10-year follow-up costs after prostate cancer treatment with curative (surgery, radiotherapy) and non-curative intent (hormone, androgen deprivation) per patient in Germany in 2000, 2008, and 2015.

**Methods:**

Prostate cancer follow-up recommendations were extracted from the European Association of Urology guidelines from 2000 to 2015. Per patient costs were calculated with a detailed micro-costing approach considering direct and indirect medical expenses. Input parameters were derived from expert interviews, literature research, and official scales of tariffs. Costs for insurers, providers, and payers were included to estimate societal costs.

**Results:**

Mean 10-year follow-up costs per patient after treatment with curative intent amounted to EUR 4415 in 2000, EUR 4224 in 2008 (*p* < 0.001), and EUR 5159 in 2015 (*p* < 0.001). Costs after hormone therapy with metastasis cumulated to EUR 10,846 in 2000, EUR 9818 in 2008 (*p* < 0.001), and EUR 11,978 in 2015 (*p* < 0.001). While insurers covered 37% of costs in 2000 (EUR 1664), only 23% of costs were reimbursed in 2015 (EUR 1195; *p* < 0.001). Cost sources mainly included consultations (55%), transportation (18%), and imaging (27%).

**Conclusion:**

Early detection and advances in prostate cancer treatment increased 10-year survival rates beyond 80% in Germany, ultimately expanding the number of survivors requiring follow-up. Statutory insurers reacted by decreasing the reimbursement rates to reduce per patient cost by up to 46%. Consequently, the economic burden was mainly shifted to payers and providers.

**Implications for Cancer Survivors:**

Equitable and effective follow-up schedules covered by insurance funds are necessary to care for prostate cancer patients.

## Introduction

In Germany, 6.8% of the gross domestic product were spent on cancer in 2014—a 1% increase from 1995 [1]. Prostate cancer is currently the most common non-skin malignancy for men in Germany with an incidence of 91.6 per 100,000 inhabitants per year [1, 2]. By 2030, it is even forecasted to be the most common malignancy in Germany [3]. However, diagnostic and treatment options steadily improved increasing 10-year survival rates beyond 80% in Germany [2]. While costs for anti-cancer drugs (e.g. androgen deprivation therapy such as abiraterone acetate, apalutamide) significantly rose, a cutback in hospital care balanced the overall surge in healthcare expenditure [1].

A variety of papers investigate prostate cancer treatment options and analyse their effectiveness [4–6]. Treatment strategies can have either a curative or a palliative intent. A relapse of carcinoma might be treated by active surveillance or salvage radiation (after an initial radical prostatectomy) or salvage prostatectomy (after an initial radiotherapy). Furthermore, follow-up treatment options are frequently examined and single aspects, such as X-rays or prostate-specific antigen (PSA), are evaluated regarding their specific cost-effectiveness [7–10]. Precisely, the measurement of PSA for follow-ups has been controversially discussed as it was found to decrease mortality from prostate cancer but does not alter all-cause mortality [11–13]. Pearce et al. (2016) previously evaluated the cost of follow-up strategies across the UK [14]. He concluded that Ireland could have saved up to 26% of follow-up costs per year by implementing the European Association of Urology’s (EAU) or National Institute for Health and Care Excellence‘s (NICE) guidelines. To the best of our knowledge, there is currently no study analysing follow-up cost for prostate cancer on a patient level in Germany. Therefore, this paper examines 10-year follow-up costs from 2000 to 2015 based on the EAU recommendations. A detailed micro-costing approach considering direct and indirect medical expenses for insurers, providers, and payers is used to estimate overall societal costs.

### Prostate cancer guidelines

The EAU first issued guidelines for prostate cancer follow-up in 2000 [15]. Recommendations were subsequently updated annually [16–19]. The guideline recommends separate follow-up patterns after treatments with curative intent (radical prostatectomy or radiotherapy) and after hormonal treatments.

#### Curative intent

EAU guidelines recommended detecting treatment failures early by conducting frequent examinations during the first 2 years. Specifically, examinations were recommended in months 3, 6, 12, 18, and 24, and thereafter annually. While patients should also be monitored for PSA levels, prostate cancer may also progress without change in PSA due to differentiated cells without capacity to secret PSA. Clinical or laboratory suspicion of cancer relapse should trigger a chest X-ray, or abdominal and transrectal ultrasound examinations. Medical imaging is not recommended as a means for routine follow-ups. CT and MRI should not be used during routine follow-ups of asymptomatic patients.

#### Hormone

Patients that receive hormonal treatment should be examined 3 and 6 months after initial treatment. Thereafter, patients without distant metastasis and good treatment response should be re-examined every 6 months. In contrast, patients with distant metastases and good treatment response should be examined every 3–6 months. Patients receiving antiandrogen treatment might require more intensified follow-up patterns. Bone scintigraphy was not recommended as a routine procedure for asymptomatic patients. CT or MRI scans might be useful to find node metastases in patients with negative bone scintigraphy and elevated PSA levels. Patients that do not have an adequate hormone treatment response or a disease progression need “individualized” follow-ups.

## Methods

### Follow-up costs per patient

The EAU guidelines suggest distinct prostate cancer follow-up patterns and procedures according to the cancer stage and prior therapy. Consequently, costs were estimated for four distinct therapy types: curative, hormone therapy without metastasis, hormone therapy with metastasis, and androgen depravation therapy. While the EAU clearly proposes a lifelong follow-up, international recommendations for budget impact analysis endorse a follow-up period of 1–6 years [20, 21]. Merging these competing views, 10 years was assumed as appropriate. Cost data were calculated with a detailed micro-costing approach derived from patient level data to ensure a high level of precision. The developed costing approach estimates follow-up costs from all three perspective—payers, providers, and insurers—to then estimate the overall societal impact in 2000, 2008, and 2015.

#### Payer perspective

The payer perspective estimates the opportunity costs associated with prostate cancer follow-ups. Time consumption for patients was either based on literature or expert opinions. The forgone opportunity cost was calculated based on the average salary and the worked/productive hours. Calculations further accounted for the higher annual income of privately insured patients (2000: EUR 39,574; 2008: EUR 48,150; 2015: EUR 54,900) relative to statutory insured patients (2000: EUR 30,612; 2008: EUR 37,236; 2015: EUR 43,344). Overall, the majority of patients (88%) are insured through the statutory insurance scheme, while only 12% take out private health insurance.

#### Provider perspective

The provider perspective calculates the opportunity costs for health care providers (e.g. physicians and medical assistants) with prostate cancer follow-ups. In Germany, the provider perspective is relevant since budgeting and reimbursement restrictions lead to a discrepancy between the provider’s possibility to bill medical services and actual performed medical activities. Time consumption was again based on previous literature and expert interviews. The forgone opportunity cost was calculated based on the average salary and the worked/productive hours [22–24]. Opportunity costs per hour of patient contact rose throughout the examined period for both physicians (2000: EUR 203.20; 2008: EUR 230.25; 2015: EUR 305.37) and medical assistants (2000: EUR 46.13; 2008: EUR 40.28; 2015: EUR 59.32).

#### Insurer perspective

The insurer perspective considers the bill that the provider hands to the insurer. Due to Germany’s dual insurance system, a separate costing approach was considered for both the social health insurance (SHI) and the private health insurance (PHI). SHI funds are charged according to reimbursement rates published in a catalogue (“*Einheitlicher Bewertungsmaßstab – EBM*”) [25–27]. Similarly, private insurance firms are billed based on established reimbursement rates in a distinct catalogue (“*Gebührenordnung für Ärzte – GOÄ*”) [28]. Reimbursement rates for relevant services from the EBM and GOÄ catalogue were extracted for 2000, 2008, and 2015 (Table 1).

**Table 1:** Input parameters. Reimbursement rates for private and statutory health insurance were extracted from the respective reimbursement catalogue: “*Gebührenordnung für Ärzte (GOÄ)*” and “*Einheitlicher Bewertungsmaßstab (EBM)*” [25–28]. Time consumption for physicians, medical assistants, and patients was extracted from relevant literature and confirmed in expert interviews

	Private health insurance: GOÄ (EUR)			Social health insurance: EBM (EUR)			Time consumption (minutes)						
	2000	2008	2015	2000	2008	2015	Physician	Ref.	Assistant	Ref.	Patient	Ref.	Distribution
Physician-patient consultation	20.10	20.10	20.10	9.86	20.33	20.34	7.6	[31]	-		7.6	[31]	Gamma
Physical examination	34.86	34.86	34.86	11.90	10.75	11.61	15.8	[31]	-		15.8	[31]	Gamma
Prostate sonography	26.81	26.81	26.81	14.88	8.23	8.94	20.0	[32]	-		20.0	[32]	Gamma
Prostate sonography (incl. biopsy)	294.91	294.91	294.91	30.88	36.79	39.77	40.0	[33]	-		40.0	[33]	Gamma
PSA (incl. blood taking)	24.30	24.30	24.30	11.00	5.60	4.80	-		5.0	[31]	5.0	[31]	Normal
Testosterone levels	-	-	20.40	-	-	5.00	-		0.5	[31]	1	[31]	Normal
Blood marker (Hb, Crea, AP)	9.38	9.38	9.38	0.92	0.90	0.90	-		5.0	[31]	5.0	[31]	Normal
Pelvic MRI	461.64	461.64	461.64	91.14	157.58	170.48	30.0	[34, 35]	45.0	[36]	30.0	[34, 35]	Normal
Digital rectal examination	37.54	37.54	37.54	31.25	8.06	8.73	4.0	[27]	-		4.0	[27]	Gamma
Travel time	-	-	-	-	-	-	-		-		60.0	[31]	Gamma
Waiting period	-	-	-	-	-	-	-		-		45.0	[37]	Gamma
Travel cost (km)	-	-	-	-	-	-	-		-		40.0	[38]	Gamma
Parking costs	-	-	-	-	-	-	-		-		45.0	[39]	Gamma

#### Cost calculation

Follow-up costs were estimated based on a patient-level micro-costing approach. The frequency of recommended consultations, examinations, and diagnostic tests was extracted from EAU guidelines. Thereafter, the opportunity costs for physicians and patients, resulting from forgone time consumption for follow-ups, were calculated alongside the resulting expenditure bill for the insurance (Table 1). The hospital & community health services index was used to adjust all healthcare-related costs for inflation [22–24]. Cost progression was compared across years, stakeholder, and resource use.

#### Sensitivity analysis

We conducted a probabilistic sensitivity analysis to account for variations in the length of physician-patient consultations, examinations, and diagnostics. Therefore, provider’s and payer’s time consumption parameters were drawn by random sampling from their defined distribution (Table 1). In Germany, reimbursement rates are independent of time consumption and were hence not included in the sensitivity analysis. The analysis estimated the costs for 1000 patients per treatment cohort and year. Cost data were expressed as means ± standard deviations. For the two-factorial analysis of variance, ANOVA with Dunnett’s test was applied. A two-tailed probability value <0.05 was considered significant.

### Budget impact analysis

Budget impact analysis is employed to assess changes in healthcare expenditure following a decision to reimburse new treatments or related policies on a population level [21]. Consequently, we estimated the budget impact on healthcare expenditure in Germany resulting from revised EAU guidelines and amended reimbursement rates, as well as shifting prostate cancer incidence and survival. Yearly prostate cancer incidence rates were extracted from the GENESIS database [2]. Follow-up costs per patients were based on the previously calculated cumulative societal perspective. Survival rates and the split between treatment types (curative, hormone, and androgen deprivation) were extracted from literature [29, 30]. A deterministic sensitivity analysis was performed to scrutinize uncertainties surrounding point estimates of cancer incidence, follow-up cost per patient, discount rate, and 10-year cancer survival on the healthcare budget. Each point estimator was univariately varied at a time according to the underlying 95% confidence interval.

## Results

### Follow-up costs per patient

**Fig. 1:**
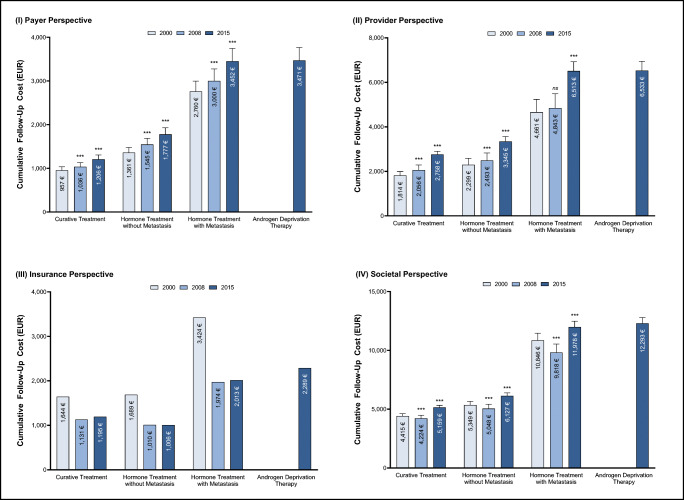
Cumulative 10-year prostate cancer follow-up costs (EUR) per patient by initial treatment type from the (**I**) payer, (**II**) provider, (**III**) insurance, and (**IV**) societal perspective. All costs were inflation adjusted with the hospital & community health services index [22–24]. *p* values compared to year 2000: *p <* 0.05 (*), *p <* 0.01 (**), *p <* 0.001 (***). Bars show standard deviations. Insurance bills do not possess standard deviations because fixed reimbursement rates were extracted from the official scales of tariffs

Estimated 10-year prostate cancer follow-up costs ranged from EUR 4224 to EUR 12,293 per person (Fig. 1). However, cost progression diverged between payers, providers, and insurers from 2000 to 2015.

#### Payer perspective

Ten-year follow-up costs for payers increased throughout the examined period (Fig. 1I). Follow-up costs after curative treatment increased from EUR 957 in 2000 to EUR 1206 in 2015 (*p* < 0.001). The incline was even steeper for follow-up schedules after hormone treatment without metastasis (2000: EUR 1361; 2015: EUR 1777; *p* < 0.001) and with metastasis (2000: EUR 2760; 2015: 3452; *p* < 0.001).

#### Provider perspective

The calculated 10-year follow-up costs constantly rose from 2000 to 2015 for providers (Fig. 1II). Costs after treatment with curative intent surged from EUR 1814 in 2000 to EUR 2758 in 2015 (*p* < 0.001). Follow-up costs after hormone treatment without (2000: EUR 2229; 2015: 3344; *p* < 0.001) and with (2000: EUR 4661; 2015: EUR 6513; *p* < 0.001) metastasis behaved in a similar pattern.

#### Insurance perspective

Overall, 10-year follow-up costs decreased from 2000 to 2008 and then remained at the same level until 2015 (Fig. 1III). For example, follow-up costs after curative treatment decreased from EUR 1644 in 2000 to EUR 1131 in 2000 and only slightly increased to EUR 1195 until 2015. Costs for SHI and PHI follow the same underlying pattern. However, the cost reduction was more drastic for the statutory insurance relative to the private insurance. Compared to 2000, statutory insurance costs across all cancer entities declined by 46% in 2008 and 44% until 2015. In contrast, costs for private insurers only shrunk by 8% in 2008 and 8% until 2015. Consequently, costs were 2.1× (2000), 3.5× (2008), and 3.5× (2015) higher for the private compared to the statutory insurance.

#### Societal perspective

**Fig. 2:**
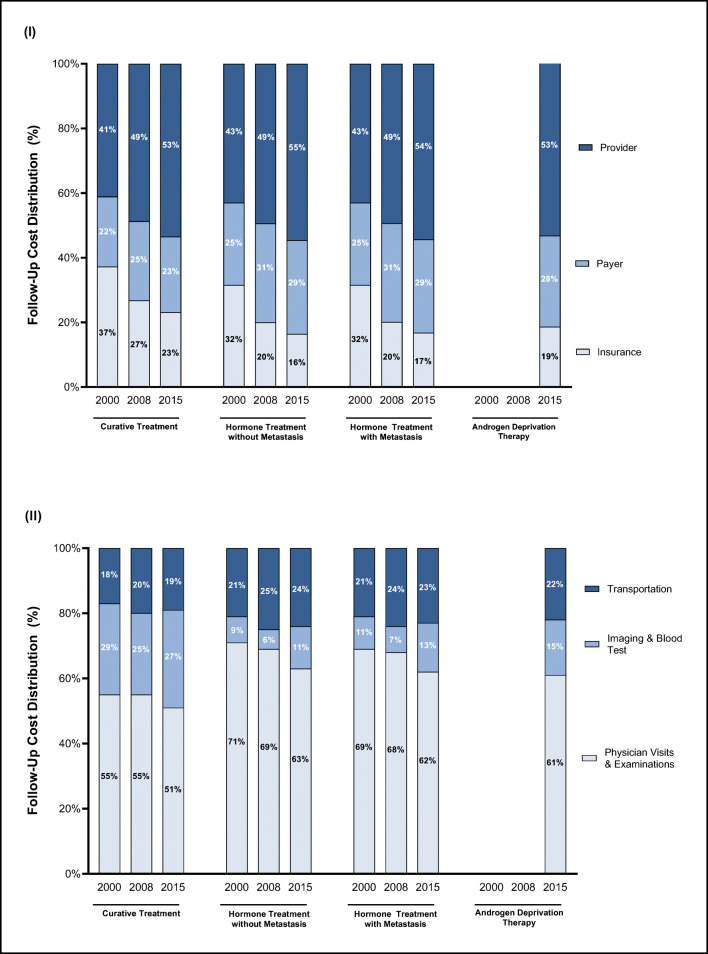
Cumulative 10-year prostate cancer follow-up cost distribution by initial treatment type for (I) perspective and (II) resource use

Taking a societal perspective, 10-year follow-up costs decreased from 2000 to 2008 and increased from 2008 to 2015 across all examined treatment types (Fig. 1IV). Follow-up costs after treatment with curative intent declined from EUR 4415 in 2000 to EUR 4224 in 2008 (*p* < 0.001) to then increase again to EUR 5159 in 2015 (*p* < 0.001). Costs followed a similar pattern after hormone therapy treatment. The decline in 2008 can mainly be attributed to the decreased costs for insurance funds (Fig. 2I). While insurance funds incurred approximately one-third of follow-up costs in 2000, this share steadily declined to approximately 20% in 2015. Consequently, most of the burden was shifted to providers and payers, which incurred more than two-thirds of follow-up costs in 2015.

Follow-up costs after hormone treatment with metastasis were significantly higher than follow-up costs after hormone treatment without metastasis across all years. The EAU recommends more frequent follow-up intervals for prostate cancer in the metastatic state to detect recurrences. Therefore, more costly consultations and examinations are required for patients with metastasis. Similarly, costs after newly introduced androgen deprivation therapy were significantly greater compared to costs after curative intent (*p* < 0.001). Patients after androgen deprivation therapy are also recommended to adhere to shorter follow-up time intervals.

Costs were also examined by resource use (Fig. 2II). Resources were mainly used for physical examinations (more than 55% of costs) and transportation (ca. 20% of costs). Imaging, such as X-ray, CT, or MRI, increased from 20% in 2000 to 26% in 2015 for follow-up costs after curative intent. Meanwhile, examination costs decreased (curative: 55% to 51%; hormone with metastasis: 71% to 63%; hormone without metastasis: 69% to 62%).

### Budget impact analysis

**Fig. 3:**
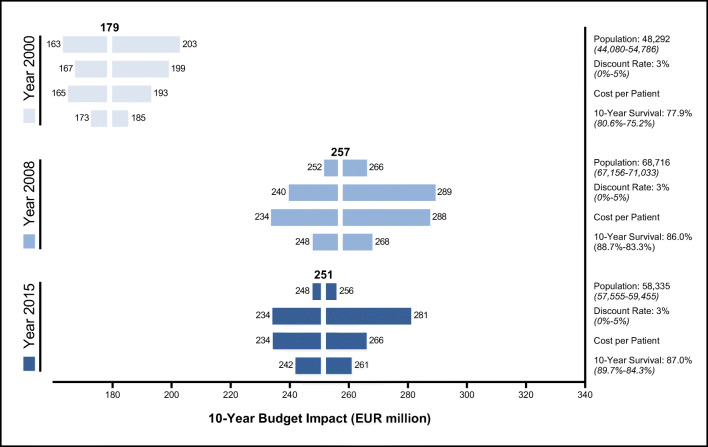
10-year budget impact analysis for prostate cancer follow-up costs (EUR million) in 2000, 2008, and 2015. Brackets represent the 95% confidence interval used to univariately vary each point estimator. Follow-up costs per patients were varied by estimations displayed in Fig. 1

The 10-year budget impact cumulated to EUR 179 million in 2000, EUR 257 million in 2008, and EUR 251 million in 2015 (Fig. 3). A deterministic sensitivity analysis was conducted by varying the follow-up population, discount rate, follow-up costs, and 10-year survival rates. Variations in the follow-up population, which rose from 48,292 (2000) to 68,716 (2008) and 58,335 (2015), altered the estimated budget impact by 13.4% (2000), 3.5% (2008), and 2.0% (2015). The budget impact was also subject to fluctuations in per patient follow-up costs estimated in Fig. 1: 7.8% (2000), 26.9% (2008), and 6.0% (2015). Uncertainty surrounding the 10-year survival rates only caused minor changes in the estimated healthcare expenditure by 3.4% (2000), 4.3% (2008), and 4.0% (2015). However, the overall prostate cancer 10-year survival rates rose from 77.9% in 2010 to 87.0%, ultimately increasing the number of cancer survivor requiring follow-up.

## Discussion and conclusion

Cancer follow-up costs are important to insurers, payers, and providers. Results show that all three stakeholders incurred significant economic costs. Therefore, the consideration of cancer follow-up costs should be incorporated into future research. Specifically, the burden of cancer survivorship imposes substantial direct and indirect costs on the overall healthcare system as well as patients. The overall financial burden depends on the stage of cancer and the “survivorship trajectory” after treatment [40]. Consequently, economic evaluations ought to include subsequent follow-up costs to the overall assessment of cancer treatment strategies. At the same time, there needs to be a balanced approach between sufficient evidence to justify costs and the effect on mortality during follow-up visits [41].

In 2000, around one-third of all costs was born by health insurances. In 2015, less than 25% was paid by insurances (depending on the specific cancer state). Due to a successive budgeting of medical services reimbursed by the German statutory health insurances, the whole burden of follow-up costs is mainly shouldered by patients and providers. As a result, patients and providers might be less incentivized to participate in cancer follow-ups. Research reveals that some patients do not participate during all recommended follow-ups [42]. Follow-up participation is especially influenced by “perceived symptoms, motivation, affect, provider influences, readiness for medical follow-up, and knowledge of treatment exposures” [43]. However, less than 30% of cancer survivors receive any post-treatment follow-up plans [44, 45]. Consequently, both providers and patients might face financial barriers to the enhanced economic burden.

Based on existing research, prostate cancer treatment costs in the first 6 months after treatment amount to USD 11,495, ranging from USD 2586 for watchful waiting to USD 24,204 for external beam radiation [46]. In contrast, our results indicate 10-year cumulative follow-up costs of EUR 1000 to 12,000 depending on the specific entity. As a result, follow-up costs are an expensive and resource intense part of cancer treatment alternatives. In 2015, Germany spent EUR 338 billion on neoplastic diseases, of which prostate cancer accounted for EUR 1.85 billion [47]. In comparison, we estimated a cumulative 10-year budget impact of EUR 251 million, resulting in a yearly budget impact of EUR 25 million. Therefore, our estimates suggest that follow-ups account for only 1.4% of the prostate cancer spending in Germany.

The overall budget impact is subject to several factors. First, adjustments in the statutory reimbursement rates until 2008 limited follow-up costs. However, increasing prostate cancer incidence combined with improved 10-year survival rates due to more sophisticated diagnostic and treatment options expanded the follow-up budget impact from 2000 to 2008 by EUR 78 million (+44.1%). German insurers likely anticipated the rising incidence, prevalence, and survival rates. As a result, insurers reduced reimbursement rates to maintain the overall healthcare budget. In contrast, from 2008 to 2015 declining incidence rates were offset by higher follow-up costs, resulting in a roughly constant expenditure.

Lately, primary and secondary preventions have been prioritized. Research has focused on the early detection of prostate cancer. In Germany, prostate cancer incidence significantly increased in the past years due to advances in early detection featuring annual digital rectal examination and possibly a PSA-level measurement [48], and changing lifestyle habits [2, 49]. Meanwhile, the combination of early detection and advances in treatment options halved mortality and pushed the 10-year survival rate well beyond 80% in Germany [50]. Therefore, a growing number of patients survive prostate cancer and are subsequently trapped in the follow-up phase. Necessarily, follow-up schedules must be revisited to account for this survivorship. More detailed classification of schedules and suitable patients is required to account for differential risk factors. Accordingly, overall follow-up cost could be reduced. Insurers and providers will need to find innovative solutions to overcome the substantial resource consumption demanded by this shift. The cautious design and implementation of disease management programmes for cancer follow-ups may further increase adherence to guidelines and align incentives among all stakeholders.

In summary, prostate cancer follow-ups pose a substantial economic burden to all stakeholders. Follow-up costs may vary depending on initial treatments and cancer stages. Therefore, it is recommended that follow-up expenses are included in cost-effectiveness evaluations of cancer treatments. Heins et al. (2018) explored the feasibility of general practitioner (GP)–led prostate cancer follow-ups [51]. Most patients and physicians were satisfied with this new model. However, lower paid GP-led follow-ups only marginally reduce physician and insurance-related costs, but do not impact the financial burden borne by patients. It remains uncertain how GP-led follow-ups impact the quality of care. Furthermore, outpatient cancer care delivery could be digitalized with virtual follow-ups—“e-oncology” [52]. Consequently, follow-ups might be (partly) replaced or supplemented by specialist nurses, telephone consultation, or digital applications. Potential reimbursement of digital applications under Digital Healthcare Act (DVG), ratified in 2020, provides the conceptual framework in Germany [53]. While Germany may still lack the digital literacy and infrastructure for e-oncology [54], such applications might improve physician-patient relations, smoothen financial burdens faced by patients and providers, and erase inequalities in care quality and access by decreasing opportunity and travel costs for patients and providers.

Our findings inform decision makers about the financial cost borne by patients and providers as well as a shift in costs covered by statutory health insurers in Germany. This shift in the economic burden from insurers to patients and providers may disincentivize adherence to follow-up guidelines and thereby adversely impact health outcome—especially for the poorer population not covered by private insurance.

### Strengths and limitations

The strengths of this paper include the detailed micro-costing approach, the consideration of all three perspectives, and the evaluation of various treatment options (curative vs. hormone therapy vs. ADT). First, direct and indirect medical costs were included for insurers, providers, and patients. Second, distinct follow-up costs for different treatment options and cancer stages inform cost-effectiveness evaluations of prostate cancer treatment options. Based on literature review, this is the first study estimating prostate cancer follow-up costs in Germany from 2000 to 2015.

There are limitations present. First, follow-up costs are empirically calculated and therefore not validated with clinical studies. Furthermore, the assumed “uptake” of 100% of guideline recommendations might not be realistic. Firstly, some physicians might not follow guideline recommendations due to several barriers. Secondly, some cancer patients might not participate in cancer follow-ups. Adherence to follow-up is generally related to patient education, availability of transportation services, and clinical efficiency [55]. Physicians in Germany could be encouraged to adhere to follow-ups by establishing extra-budgetary reimbursement incentives. A reduction of opportunity costs by limiting physical visits or partially reimbursement of transportation costs could further incentivize patients to adhere to follow-up schedules. Finally, the design and implementation of disease management programmes for cancer follow-ups could provide both patients and physicians with a financial and structural incentive to adhere to guidelines.
